# Interleukin-22 Mediates Early Host Defense against *Rhizomucor pusilluscan* Pathogens

**DOI:** 10.1371/journal.pone.0065065

**Published:** 2013-06-17

**Authors:** Wei Bao, Lei Jin, Hai-jing Fu, Yong-nian Shen, Gui-xia Lu, Huan Mei, Xin-zhi Cao, Hong-sheng Wang, Wei-da Liu

**Affiliations:** 1 Jiangsu Key Laboratory of Molecular Biology for Skin Diseases and STIs, Department of Mycology, Institute of Dermatology, Chinese Academy of Medical Sciences and Peking Union Medical College, Nanjing, China; 2 Department of Stomatology, Jinling Hospital, Medical School of Nanjing University, Nanjing, China; 3 Jinling Hospital, Medical School of Nanjing University, Nanjing, China; 4 Department of Medical Oncology, Jinling Hospital, Medical School of Nanjing University, Nanjing, China; 5 Bayi Hospital, Nanjing, China; 6 Department of Medical Imaging, Jinling Hospital, Clinical School of Medical College, Nanjing University, Nanjing, China; University of California, Riverside, United States of America

## Abstract

**Background:**

In recent years, the fungal infectious disease zygomycosis has increased in incidence worldwide, especially among the immunodeficient population. Despite the rates of zygomycosis-related death and deformation being very high, the mechanism(s) by which the fungal pathogens cause these severe manifestations remain unknown.

**Methods:**

Using the associated *Rhizomucor variabilis* species, which can selectively induce cutaneous zygomycosis in otherwise healthy individuals, we investigated the host mechanisms of infection-related responses, including cytokine and chemokine expression as well as contributions of particular T cell subsets. siRNA specifically targeting IL-22,IL-17 and IFN-γ were used to down-regulate expression of those molecules.

**Results:**

In mouse models of infection, IL-22 was implicated in development of *Rhizomucor spp.*-induced skin lesions. In cultured human peripheral blood monocytes, *R. pusilluscan*, which is often found in immunodeficient patients, induced the production of IL-22, while *R. variabilis* did not. Moreover, *Rhizomucor spp.*-induced secretion of Il-22 from CCR6^+^CCR4^+^CCR10^+^ cells was down-regulated by knockdown of IL-22 related signaling receptors, RORC and ARH.

**Conclusion:**

Our data strongly suggest that avoidance of IL-22 may be one mechanism by which mucor species produce morbidity and mortality in infected individuals.

## Introduction

In recent years, the incidence of zygomycosis has increased in the immunodeficient population [1], with the most common manifestations involving the sinuses, pulmonary system, and skin (cutaneous). Primary cutaneous zygomycosis (PCZ) was first reported in 1929, and since then sporadic cases have been reported across the world [2]. Several pathogenic fungal species have been detected in PCZ patients and implicated as causative agents of this widespread but largely unknown disease, including *Rhizopus spp*., *Absidia corymbifera*, *Mucor spp.*, *Rhizomucor pusillus*, *Cunninghamella bertholletiae*, *Saksenaea vasiformis*, and *Apophysomyces elegans* [3]. In addition, we and others have reported cases of *Rhizomucor variabilis*-related PCZ in China [4]. *R. variabilis* represents a unique and interesting pathogen of zygomycosis, as it can induce disease in otherwise healthy individuals with intact immune systems. As such, it may represent a useful model to elucidate the host immunopathobiology and identify manipulable factors for therapeutic strategies to defend against mucor invasion in PCZ.

The well-studied cytokine, interleukin (IL)-22, plays a key role in host defense against pathogenic bacteria, viruses, and fungi. Indeed, studies of various bacterial infections in mouse models have highlighted a protective role for IL-22 [5], [6]. Recent reports indicated that diseases associated with chronic overgrowth of the gut-localized yeast *Candida albicans* were characterized by down-regulated expression of IL-22 and IL-17 [7]. Considering the roles of IL-22 in human immune response to such a wide variety of pathogens suggests the potential of a likely protective role against mucor infection.

IL-22, a member of the IL-10 family of cytokines, signals through the heterodimer receptor complex consisting of the IL-22 receptor (IL-22R) subunit paired with IL-10Rb subunit [8]. Whereas IL-10Rb is ubiquitously expressed, IL-22R is expressed specifically on epithelial cells [9], in which it is believed to mediate epithelial innate immunity [10]. In the human, IL-22 is produced by leukocytes, with particularly high levels from the CD4^+^ effector T cell subset of T helper type 22 (Th22) cells [11]. In the mouse, IL-22 is primarily produced by Th17 cells, wherein its expression is controlled by the Th17 lineage-specific transcription factor RORγt [12], [13]. Transduction of the human form of RORγt, RORC, into naive human cells does not stimulate production of IL-22, which suggests a key difference between IL-22 production in humans and mice [14].

The aryl hydrocarbon receptor (AHR) is another host factor that has been defined as a mediator of IL-22 expression. AHR interacts with many physiologic ligands, including the tryptophan metabolites (such as 6-formylindolo[3,2-b]carbazole), as well as pathologic ligands, including environmental toxins (such as 2,3,7,8-tetrachlorodibenzo-p-dioxin) [15]. Agonists of AHR have been shown to increase expressions of both IL-17 and IL-22, indicating its role in Th17 cell function [16], [17]. Further studies of AHR-deficient mice revealed that T cells require AHR to produce IL-22 [9].

The role of IL-22 in host defense against bacterial and viral infections has been extensively studied [17]. In the skin, IL-22 has been shown to induce antimicrobial peptides, promote keratinocyte proliferation, and inhibit differentiation. Thus, IL-22 is likely to play a role in wound healing processes and/or immunity-related mechanisms [18], making it an intriguing candidate for the immunopathobiology of fungal infections, such as mucormycosis.

In this study, we investigated the potential role of IL-22 in *Rhizomucor spp.* infections using *in vivo* mouse models and *in vitro* human peripheral blood monocyte cell (PBMC) culture models of *R. pusillus* and *R. variabilis* infection. Specifically, we profiled the particular T cell subsets related to each fungal species and the interacting cytokine and chemokine factors to gain insights into the differential mechanisms underlying the two. Collectively, we found that IL-22 might play an important role in host defense against *Rhizomucor spp*. infections, in which *R. variabilis* may establish infection in otherwise healthy people through avoiding induction of IL-22.

## Materials and Methods

### *Rhizomucor spp.* mouse infection model

Seven-week old BALB/c mice, weighing 22–24 g, were housed at 22°C under a 12-h light-dark cycle and with *ad libitum* access to food and water. The mice were assigned to five groups(n = 5/per group) for enzyme-linked immunosorbent assay (ELISA) detection at various time points, four groups(n = 10/per group) for detection of the effects of small interfering (siRNA) targeting IL-22, IL-17, and IFN-γ, respectively, and six groups(n = 100/per group) for lesion percent detection. All procedures involving the animals were carried out in accordance with the recommendations of the Council of European Communities Directive 86/609/EEC (24 November 1986) and were approved by the Ethics Committee of the Chinese Academy of Medical Sciences and Peking Union Medical College.

For *R. pusillus* and *R. variabilis* infection, *R. pusillus*(B63) and *R. variabilis* strains(B50) were obtained from the National Fungi Strain Reserve Center (Nanjing, China) and cultured on Sabouraud dextrose agar supplemented with 0.02% chloramphenicol for seven days at 35°C. Spores of the respective strains were harvested by washing the agar surface with sterile saline containing 0.05% Tween 80. The resultant spore suspensions were filtered through nylon filters (11 µm pore size), counted via hemocytometer, and stored at 4°C for no more than 24 h. Meanwhile, spore viability was determined by plating serial dilutions of the spore suspension (in saline with 0.05% Tween 80), incubating for 24 h at 35°C, and counting the colony forming units (CFU). For animal infection, the spore suspension was adjusted with saline to 10^6^ spores/0.1 mL aliquots for single injections into each mouse via intradermal inoculation into the shaved skin of the side near a flank. Biopsies were obtained from infected mice on post-infection days 1, 2, 3, 4 and 5. In addition, the mice infected with *R. pusillus* were divided into six groups of 100 for respective injections with siRNA targeting IL-22, IL-17, or IFN-γ, or unrelated (control) siRNA. The control siRNA targeted enhanced-green fluorescent protein (EGFP). Percent of lesion occurrence was observed in six groups of mice (n = 100/per group) injected respectively with siRNA targeting IL-22, IL-17, or IFN-γ, or unrelated control siRNAs corresponding to each experimental group.

The siRNA were injected into the mice at day 2 before infection with *R. pusillus*, and the knockdown efficiency was analyzed in four different groups (n = 10/per group): unrelated siRNA(controls); siRNA targeting IL-22; siRNA targeting IL-17; siRNA targeting IFN-γ, and biopsies were obtained at post-siRNA injection days 4 (for IL-22 and IFN-γ analyses) and 5(for IL-17 analysis).

### Collection of lesional punch biopsy skin specimens

Lesional 8-mm punch biopsies from infected mice were obtained and divided into two groups for processing. The first group of samples was incubated in RPMI-1640 medium (Gibco, Grand Island, NY, USA) containing dispase II (2.4 U/mL; Roche, Indianapolis, IN, USA) for 1 hour at 37°C to separate the dermis., Total RNA was extracted from the dermal specimens using the TRIzol Reagent (Invitrogen, Carlsbad, CA, USA) and stored at −20°C for subsequent use in quantitative real-time reverse transcription-polymerase chain reaction (qRT-PCR) analysis. The second group of samples was homogenized with a Pro200 series homogenizer (Pro Scientific, Oxford, CT, USA) for subsequent analysis of protein products by ELISA kits to detect levels of IL-22, IL-17, and IFN-γ (Aushon Biosystems, Billerica, MA, USA).

### qRT-PCR

Total RNA was applied as template to the reverse transcription reaction using SuperScript™ II Reverse Transcriptase (Invitrogen). The resultant cDNA was detected using a SYBR Premix Ex*Taq*™ kit (TaKaRa, Shiga, Japan). Each cDNA sample was added (5 µL) to a PCR reaction mixture containing 2 µL of 10× SYBR Green I PCR buffer (Invitrogen), 1.2 µL of 50 mM MgCl_2_, 0.4 µL of 10 mM GeneAmp dNTP mix (Applied Biosystems Inc. (ABI), Foster City, CA, USA), 0.8 µL of 10× SYBR Green I (Invitrogen), 0.1 µL of 5 U/µL Platinum *Taq* DNA Polymerase (Invitrogen), and 4 µL of 2.5 µM primer mix (forward and reverse primers for target genes). PCR reactions were carried out in an ABI 7500 real-time PCR system using the following thermal cycling conditions: initial denaturation at 95°C for 1 min, followed by 40 cycles of 95°C for 15 s, 60°C for 30 s, and 72°C for 40 s. A melting curve was generated at the end of the PCR run over the range of 55–95°C by increasing the temperature stepwise by 0.5°C every 2 s. Each qRT-PCR reaction was carried out in duplicate. Gene-specific amplification was confirmed by resolution of a single band on a 2% agarose gel stained with ethidium bromide. The expression of target genes was normalized to the control reference housekeeping gene, GAPDH, using the ΔΔC_T_ method: ΔΔC_T_ = ΔC_T sample_−ΔC_T reference_. Primers used in this section were listed in Table 1.

**Table 1:** Primers used in this study.

Gene name	Primers	
	Forward	Reverse
IL-22	5′-TCCGAGGAGTCAGTGCTAAA-3′	5′-AGAACGTCTTCCAGGGTGAA-3′
IL-17	5′-GCTCCAGAAGGCCCTCAGA-3′	5′-CTTTCCCTCCGCATTGACA-3′
IFN-γ	5′-GATCCTTTGGACCCTCTGACTT-3′	5′-AGACAGTGATAAACTATAAATGAGCG-3′

### Preparation of PBMCs

PBMCs were isolated from blood of healthy human volunteers using the standard method of Ficoll-Hypaque density gradient centrifugation. The PBMCs were then resuspended at a concentration of 2×10^6^/mL in RPMI-1640 medium supplemented with 10% fetal calf serum (Sijiqing, Hangzhou, China), 100 U/mL penicillin, 100 mg/mL streptomycin, 50 mM 2-mercaptoethanol, and 2 mM L-glutamine (all from Gibco). The portion of our study involving human samples was approved by the Medical School Review Board of the Institute of Dermatology at the Chinese Academy of Medical Sciences and Peking Union Medical College. The monocyte-derived DCs were generated by culturing human monocytes in medium supplemented with granulocyte-macrophage colony-stimulating factor (40 ng/mL) and IL-4 (40 ng/mL). The DCs were then incubated overnight with *R. pusillus* or *R. variabilis*(MOI of 50∶1), washed twice in PBS, and mixed at a ratio of 1∶1 with PBMCs or CD4^+^ or CD8^+^ T cells. After three days of incubation, the supernatants were harvested to analyze production of IL-22 by ELISA and the cells were subjected flow cytometry to analyze the expression of intracellular cytokines.

### Flow cytometry

Monocolonal fluorescent antibodies against human CD4 and CD8 were purchased from BD Biosciences (Franklin Lakes, NJ, USA). Antibodies against IL-17 and IL-22 were purchased from Antigenix (Huntington Station, NY, USA) and eBioscience (San Diego, CA, USA), respectively. Propidium iodide (PI) was purchased from Invitrogen and used to eliminate dead cells (PI-positive) prior to immunotyping. The CD4^+^ T cells were identified by their scatter profile as PI^−^CD45^+^CD3^+^CD4^+^. For some samples, CCR6 expression was also evaluated on CD4^+^ T cells. All samples were analyzed on an LSR II System flow cytometer (BD Biosciences), and the data were analyzed using FlowJo software (version 8.2; Tree Star).

### Knockdown with siRNA

*In vivo* silencing of genes of interest was carried out by siRNA delivery through tail vein injection as previously described [19]. The siRNA targeting sequences were: mIL-22: CCATACATCGTCAACCGCA; mIL-17a: CCTCAAAGCTCAGCGTGTC; and mIFN-γ: GACTCTTTGAAGTCTTGAA. Enriched CD4^+^ T cells were transiently co-transfected with RORC-specific, AHR-specific, or non-targeting siRNA with SmartPool reagents (all from Dharmacon, Lafeyette, CO, USA) according to the manufacturer's protocol. At 48 h after transfection, knockdown efficiency was analyzed by qRT-PCR.

### Statistical analysis

Multiple comparisons were carried out by ANOVA analysis. A *p-*value of <0.05 was considered significant.

## Results

### *R. pusillus*, but not *R. variabilis*, infection stimulates IL-22 production

**Figure 1:**
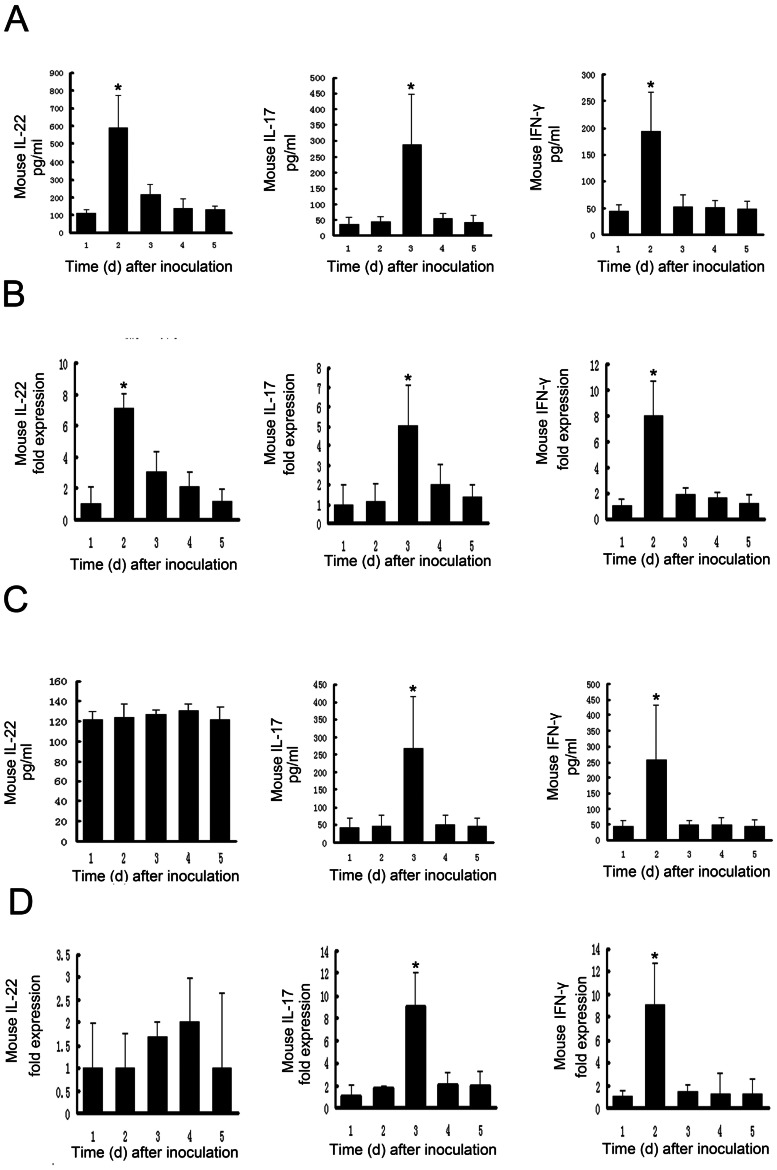
Differential cytokine profiles induced by *R. pusillus* and *R. variabilis* infections. (A) Detection of IL-22, IL-17, and IFN-γ production of protein from skin biopsies of *R. pusillus* lesions by ELISA, *P<0.05. (B) mRNAs of IL-22, IL-17, and IFN-γ from skin biopsies of *R. pusillus* lesions were detected by qRT-PCR. qRT-PCR data are presented as averaged(n = 5/per group),GAPDH-normalized fold-change versus the PBS-injected sample with the greatest ΔCt, *P<0.05. (C) Detection of IL-22, IL-17, and IFN-γ production of protein from skin biopsies of *R. variabilis* lesions by ELISA, *P<0.05. (D) mRNAs of IL-22, IL-17, and IFN-γ from skin biopsies of *R. variabilis* lesions were detected by qRT-PCR and *R. variabilis* lesions. qRT-PCR data are presented as averaged(n = 5/per group), GAPDH-normalized fold-change versus the PBS-injected sample with the greatest ΔCt, *P<0.05.

Mice infected with *R. pusillus* showed significantly increased amounts of IL-22 production in skin lesions over time (Fig. 1A)(one-way ANOVA, F = 25.432, *p* = 0.000), but mice infected with *R. variabilis* did not (Fig. 1C) (one-way ANOVA, F = 0.669, *p* = 0.621). To further investigate the cytokine profiles during *Rhizomucor spp.* infections, we quantified IFN-γ, IL-17 and IL-22 mRNA expression by qRT-PCR. IL-22 mRNA was significantly up-regulated over time in *R. pusillus* skin lesions(Fig. 1B) (one-way ANOVA, F = 42.734, *p* = 0.000), but the increased expression in *R. variabilis* skin lesions did not reach statistical significance (Fig. 1D) (one-way ANOVA, F = 1.545, p = 0.228).

IL-17 and IFN-γ mRNA expression was significantly increased over time in response to both *R. pusillus* (one-way ANOVA, F = 11.217, p = 0.000 and F = 29.167, p = 0.000, respectively; Fig. 1B) and *R. variabilis* (one-way ANOVA, F = 16.883, p = 0.000 and F = 23.915, p = 0.000, respectively; Fig. 1D). The peak expression level was reached at days 2–3 post-inoculation for all three cytokines.

### IL-22 is critical for host defense against *R. pusillus*

**Figure 2:**
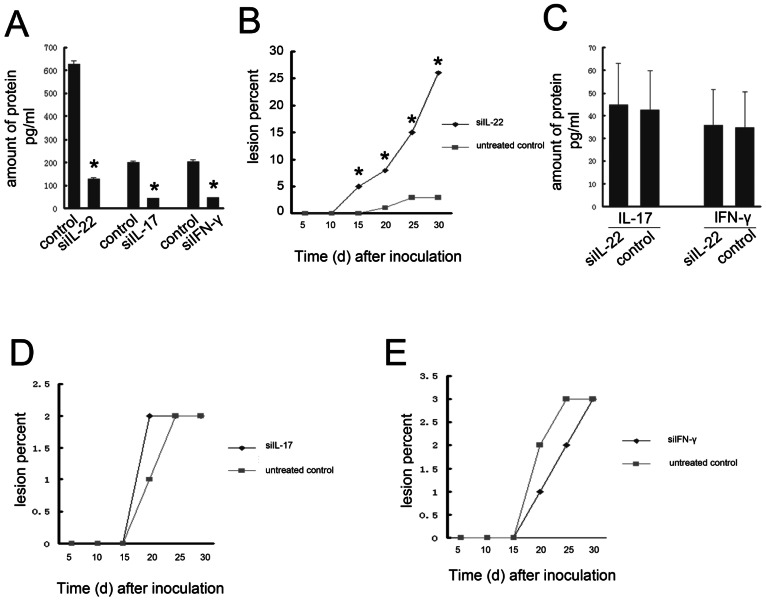
siRNA-mediated silencing of IL-22 sensitizes mice to *R. pusillus*-induced skin damage. (A) The expression of IL-22, IL-17, and IFN-γ was analysed by ELISA after transient transfection of targeting siRNAs, *P<0.05.(B) Mice were injected intradermally every other day for 16 days with 20 µL PBS, Rate of corresponding skin damage measured on days between siRNA injections targeting IL-22(n = 100/per group, *P<0.05. (C) Effect of siRNA-silenced IL-22 on IL-17 and IFN-γ was evaluated by ELISA, data are presented as averages (n = 10/per group) relative to expression in mice transfected with non-targeting siRNA (control). (D)Rate of corresponding skin damage measured on days between siRNA injections targeting IL-17 (n = 100/per group). (E) Rate of corresponding skin damage measured on days between siRNA injections targeting IFN-γ(n = 100/per group).

We used siRNA-mediated silencing to examine the role of IL-22 in *R. pusillus* infection *in vivo*. First, effective inhibition of the siRNA-targeted genes was confirmed by qRT-PCR (Fig. 2A) (Independent-Samples T test, t = 97.825,p = 0.000;t = 82.149, *p* = 0.000; and t = 57.127, *p* = 0.000, respectively). Among the *R. pusillus*-infected mice, 5.0% of those injected with IL-22-siRNA developed skin lesions 15 days later, and none of those injected with the control siRNA developed any outward signs of skin lesions during that same time period. Over the one-month observation period, the IL-22-siRNA group presented with significantly more skin lesions than the control group (27% vs. 3%, Chi-square, X^2^ *= 22.588, p = 0.000*)(Figure S1). The control group developed transient skin lesions, all of which were completely resolved by day 50 post-injection, as compared to the IL-22-siRNA group, which continued to suffer from obvious skin lesions long-term. The rate of skin lesions among the IL-22-siRNA group was 70% at two months after injection. In addition, the skin lesions of the IL-22-siRNA group emerged earlier than those in the control group (Fig. 2B).

Since IL-22 is expressed by T cells in concert with other potent cytokines, such as IFN-γ and IL-17, that mediate the immune response to pathogenic infections, we investigated whether interactions among these three factors played a critical role in sensitizing mice to *R. pusillus*-induced skin lesions. The expressions of IFN-γ and IL-17 were examined in skin lesions of IL-22 knockdown and wild-type control mice. The two groups of mice showed statistically similar levels of the two cytokines at six hours after inoculation (Fig. 2C)(Independent-Samples T test, t = 0.148, *p* = 0.884 and t = 0.319, *p* = 0.754,respectively), indicating that the role of IL-22 in *R. pusillus* was independent of these other T cell secreted cytokines. Moreover, the skin lesions in siRNA-silenced IL-17 and IFN-γ mice were not significantly different than those in controls (Fig. 2D, E)(Chi-square X^2^ = 0.338, *p* = 0.561).

### *R. pusillus* stimulates production of IL-22 but not IFN-γ or IL-17 in human CD4^+^ T cells

**Figure 3:**
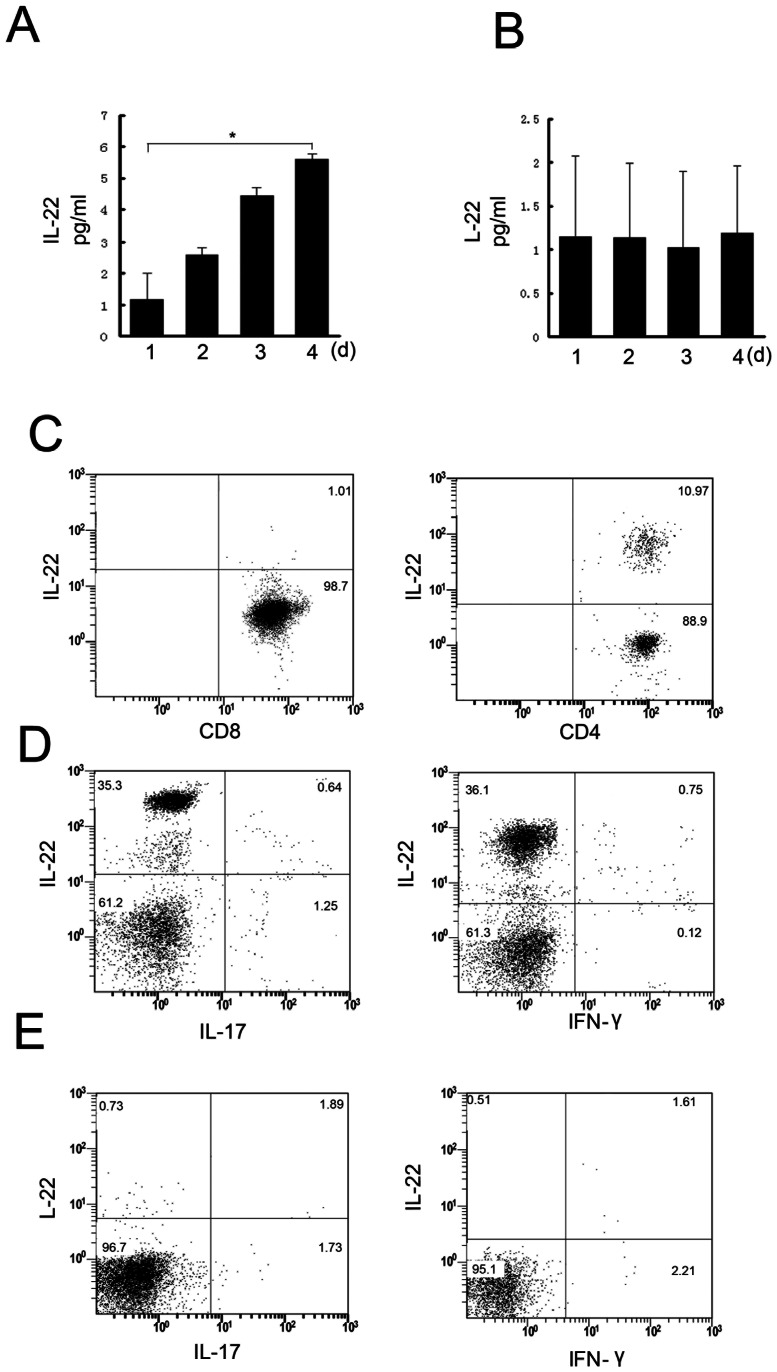
Correlation between IL-22 and Th1, Th17, and Th22 cytokines. (A) Effects of *R. pusilluscan* infection on PBMC secretion of IL-22 were determined by ELISA. Each symbol represents the mean value of triplicates from a single donor, and the horizontal bars represent the mean of all data points (n = 5/per group), *P<0.05. (B) Effects of *R. variabilis* infection of PBMC secretion of IL-22 were determined by ELISA. Data are representative of three independent experiments(n = 5/per group). (C–E) Flow cytometry of IL-17, IL-22, IFN-γ and IL-10 in CCR6^+^CCR4^+^CXCR3^−^ cells, sorted as CCR10^+^ and CCR10^+^ cells, after six days of *in vitro* expansion and restimulation with TPA and ionomycin. Numbers in quadrants indicate percent cells in each. Data are representative of three independent experiments.

To confirm our observations of IL-22 from the mouse model of infection in a human cell system, human PBMCs were stimulated with *R. pusillus* and *R. variabilis*. *R. pusillus* significantly induced IL-22, and the response was time-dependent (Fig. 3A) (one-way ANOVA, F = 91.028, *p* = 0.000). In contrast, *R. variabilis* infection produced no appreciable effect on IL-22 expression (Fig. 3B)(one-way ANOVA, F = 0.031, *p* = 0.992). T cell subpopulation analysis demonstrated that under *R. pusillus* infection conditions the CD4^+^ T cells, and not CD8^+^ T cells, were the principal expressers of IL-22 (Fig. 3C). Flow cytometric analysis of the intracellular cytokine expression in *R. pusillus*-infected CCR10^+^ and CCR10^−^ cells subsets showed that the CCR10^+^ population was enriched for IL-22^+^IL-17^−^ cells (Fig. 3D), whereas *R. variabilis* did not stimulate IL-22 production from the CCR10^+^ population (Fig. 3E).

### *R. pusillus* augments IL-22 in human CD4^+^ memory T cells through RORC and AHR

**Figure 4:**
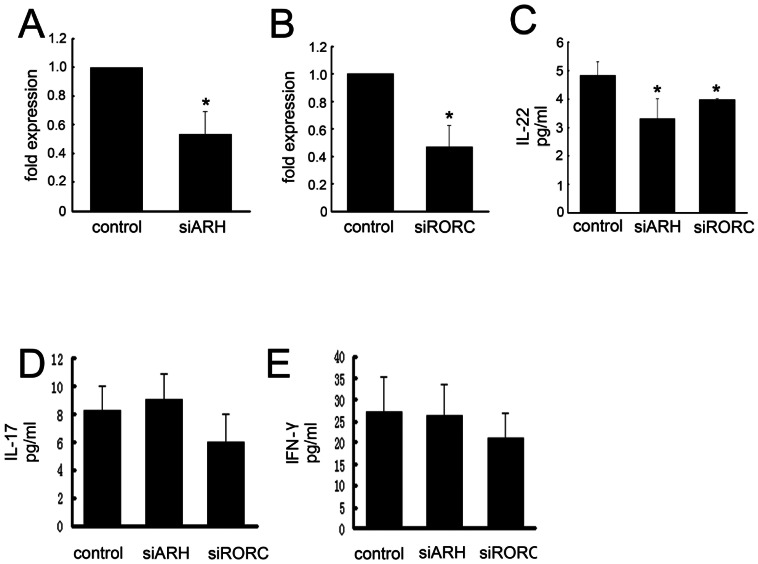
Knockdown of RORC or AHR in CD4^+^ memory T cells has different effects on the production of IL-22, IL-17, and IFN-γ. (A) qRT-PCR analysis of the AHR mRNAs in CD4^+^ memory T cells at 48 h after transient transfection of AHR-specific(n = 5/per group) , *P<0.05. (B). qRT-PCR analysis of the RORC mRNAs in CD4^+^ memory T cells at 48 h after transient transfection of RORC-specific(n = 5/per group). Data are presented relative to expression in cells transfected with non-targeting siRNA (control), *P<0.05. (C) ELISA of IL-22 cytokine secretion by siRNA-transfected CD4^+^ memory T cells(n = 5/per group) , *P<0.05. (D) ELISA of IL-17 cytokine secretion by siRNA-transfected CD4^+^ memory T cells(n = 5/per group). (E) ELISA of IFN-γ cytokine secretion by siRNA-transfected CD4^+^ memory T cells(n = 5/per group).

We transiently transfected *R. pusillus*-stimulated CD4^+^ T cells, which include the IL-17^+^ and IL-22^+^ subpopulations, with RORC-specific or AHR-specific siRNA or with a pool of non-targeting siRNAs. In all cases, 50% or more down-regulation of RORC (Independent-Samples T test, t = 6.586, *p* = 0.003) and AHR (Independent-Samples T test, t = 7.521, *p* = 0.002) mRNA was achieved (Fig. 4A, B). RORC-specific siRNA diminished the production of IL-22 at an average of 32% , while AHR-specific siRNA also substantially diminished the secretion of IL-22 (40% average inhibition; Fig. 4C) (one-way ANOVA, F = 11.338 *p* = 0.002) but did not significantly affect IL-17 production (Fig. 4D)(one-way ANOVA, F = 3.287, *p* = 0.073). Other cytokines, such as IFN-γ, were not significantly affected by any siRNA used (Fig. 4,E)(one-way ANOVA,F = 1.011, *p* = 0.393).

## Discussion

Zygomycosis has become as an increasingly important disease during the past decade. This increase has been particularly evident in hematopoietic stem cell transplant recipients and patients with hematological malignancies, cancer, receipt of organ transplant, and inherited immunodeficiencies [20]. The pathogenic fungus, *R. variabilis*, an important cause of zygomycosis, has also emerged as a frequently lethal infection in hosts without any apparent immune impairment.

*R. variabilis* infections have been reported among a heterogeneous population [4], with >50% of all infected patients having no underlying condition. However, the pathogenic mechanism of *R. variabilis* remains largely unknown. Mouse-based studies have revealed that Th17 cells co-express IL-22, which plays an important role in antimicrobial responses and autoimmune diseases [21]. Similarly, in humans, IL-22 was demonstrated to be up-regulated during defense responses against fungal infections [22].

In this study, we examined the dynamic host expression profiles of IFN-γ, IL-17, and IL-22, in response to *Rhizomucor spp.* infections. We found that all three of these cytokines are quickly induced by *R. pusillus* infection, but only IL-22 is indispensable for formation of *R. pusillus*-induced skin lesion. In addition, IL-17 signaling is dispensable for activation of the host defense against a primary challenge with *R. pusillus*, suggesting that the Th22 lineage may have evolved to mediate host defense at mucosal surfaces against extracellular fungal pathogens. This theory is supported by the previous findings that IL-22 also plays an important role in the systemic immune response against the fungus *Candida albicans* [23], as well as for clearance of intra-abdominal infection with *Escherichia coli* [24].

Previous studies of IL-17 signaling in response to specific pathogenic agents have reported findings similar to our observations of a non-essential nature. For example, IL-17 signaling is not required for host defense against primary infection with the pathogenic bacteria, *Mycobacterium tuberculosis* [25]. Albeit, that same study showed that bacille Calmette-Guerin (known as BCG) tuberculosis vaccine-induced protection requires local recruitment of IL-17–producing T cells, and that IL-17 was able to regulate TH1 cell recruitment. Subsequent studies showed that IL-17 is also required for vaccine-induced immunity against the bacteria species, *Stapholococcus pneumoniae* [26] and *Brucella pertussis* [27].

In a previous study of anti-fungal host responses, IL-22 was shown to be required in mice for effective defense against *C. albican*s [28]. In the current study, we found that the murine host response to *R. pusillus* involved induction of IL-22 and IL-17, while *R. variabilis* only stimulated production of IL-17 and not IL-22. siRNA-mediated silencing of IL-17 had no affect on the *R. pusillus*-related skin damage in the murine model system, further supporting the theory that IL-22 represents a key cytokine related to infection with this particular *Rhizomucor* species.

The Th22 cells specific for anti-*C. albicans* responses in humans were found to be prevalent in peripheral blood [29]. In the current study, we found that PBMCs from healthy individuals could produce IL-22 in response to *R. pusillus*, and not *R. variabilis*. We obtained evidence to suggest that memory IL-22-producing T cells may exert pathogen-specific functions. Specifically, in cultured PBMCs from healthy human donors *R. variabilis* induced a low level of IL-22, whereas *R. pusillus* induced a remarkably high level of IL-22. Further analysis of this subset of IL-22 overexpressing cells revealed no detectable production of IFN-γ or IL-17, ruling out Th1 or Th-17 phenotypes. It has been shown before that transduction of RORC into naive human T cells specifically induces the production of IL-17, and not IL-22 [14], which suggests that RORC is not essential for IL-22 production by human T cells. In our current study, we observed that freshly isolated CCR10^+^ IL-22-producing cells expressed RORC. Moreover, we determined that RORC-specific siRNA affected IL-22 secretion from human memory T cells, suggesting that RORC influences IL-22 production. Given the broad expression of AHR that has been demonstrated in various subsets of memory and *in vitro*–derived helper T cells, it is possible that other factors, such as RORC, may interact with AHR to mediate the regulation of IL-22.

Analysis of the chemokine receptor profile of the IL-22-producing cells contributing to the *R. pusillus* immune response revealed up-regulation of CCR4 and CCR10 expression. It is possible that these receptors and their cognate ligands serve a prominent function in skin damage induced by pathogenic fungi, or *R. pusillus* in particular. Indeed, interactions between the CCR4 cognate ligand, CCL17, and the CCR10 cognate ligand, CCL27, which are expressed by cutaneous venules and keratinocytes, respectively, have been shown to mediate recruitment of T cells to the skin [30].

In conclusion, our data clearly show the essential role of IL-22 during early host defense against fungal infections with the two common *Rhizomucor* species. Furthermore, the mechanism by which *R. variabilis* attacks otherwise healthy humans may involve an IL-22-independent pathway to avoid triggering the host's immunopathobiology.

## Supporting Information

Figure S1 **Skin lesion in the mice infected with** ***R. pusillus*** **.** Representative lesions for two mice treated with the indicated siRNA.
(TIF)
