# Supplementary figures and images for: Interleukin-22 Mediates Early Host Defense against Rhizomucor pusilluscan Pathogens
Source: PLoS One. 2013 Jun 17;8(6):e65065. doi: 10.1371/journal.pone.0065065 (PMC3684593; doi:10.1371/journal.pone.0065065)

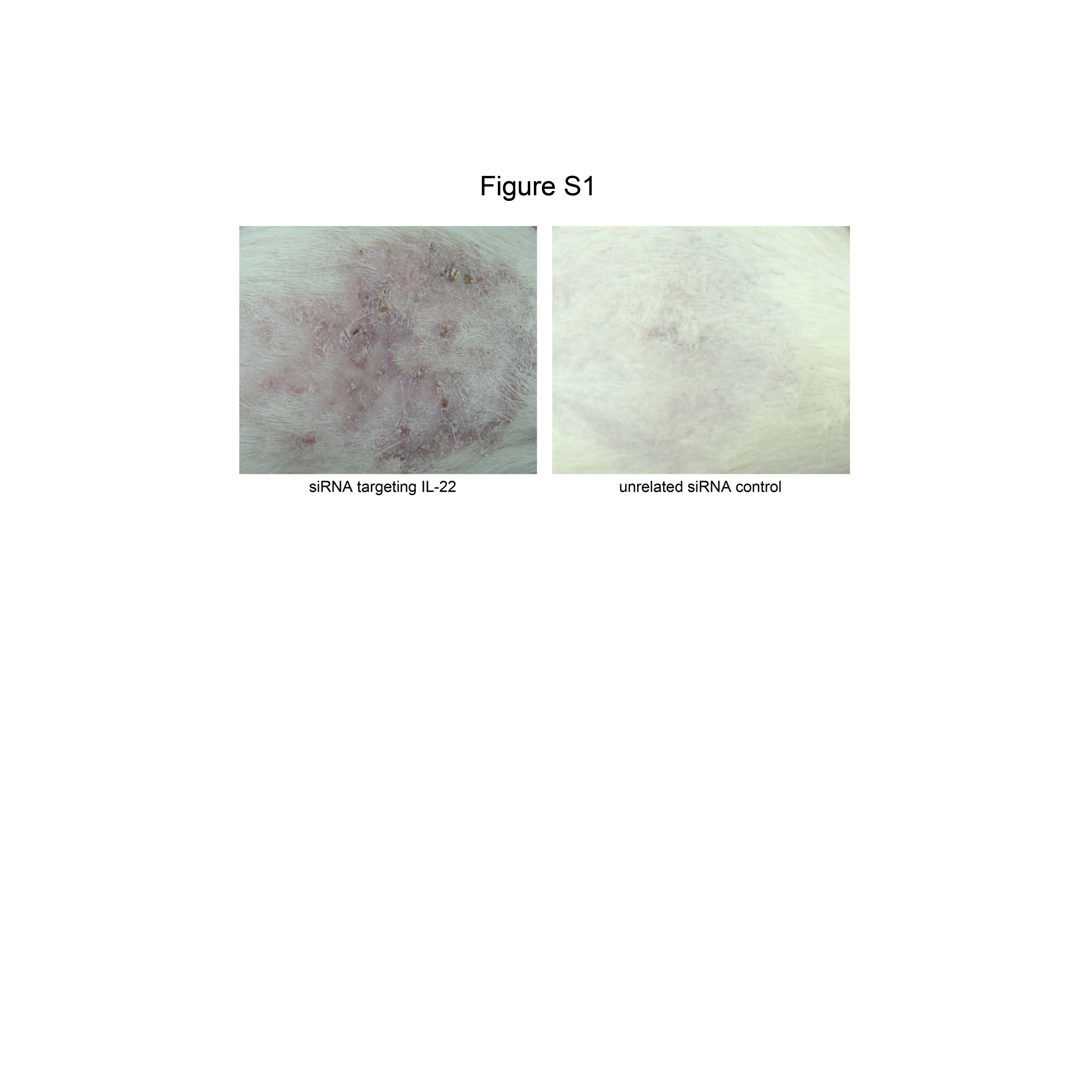

Supplement: Figure S1 — Skin lesion in the mice infected with R. pusillus . Representative lesions for two mice treated with the indicated siRNA. (TIF) [file pone.0065065.s001.tif]
